# Safety of Thread Lifting With APTOS and Energy-Based Technologies Under Artificial Intelligence-Guided High-Frequency Ultrasound

**DOI:** 10.7759/cureus.94220

**Published:** 2025-10-09

**Authors:** Daniel Perfeito, Renata Viana, Elaine Marques, Guilherme de Almeida

**Affiliations:** 1 Plastic Surgery, Olsen Almeida Dermatology, São Paulo, BRA; 2 General Surgery and Aesthetic Medicine, Artisan – Center of Excellence, São Paulo, BRA; 3 Dermatology, Olsen Almeida Dermatology, São Paulo, BRA; 4 Dermatology, Sirio Libanes Hospital, São Paulo, BRA

**Keywords:** artificial intelligence, barbed sutures, energy-based devices, facial rejuvenation, thread lifting

## Abstract

Background

Suspension threads composed of poly-L-lactic acid (PLLA) and polycaprolactone (PCL) are widely used for nonsurgical facial rejuvenation. Accurate placement within the superficial musculoaponeurotic system (SMAS) is essential to reduce risks and optimize outcomes. Real-time high-frequency ultrasound with artificial intelligence (AI) may assist procedural accuracy. The safety of combining thread lifting with energy-based technologies, performed on the same day, remains underreported.

Objective

The objective of the study was to evaluate the overall safety of APTOS PLLA/PCL threads (APTOS Excellence Visage®; APTOS LLC, Tbilisi, Georgia) placed under AI-guided high-frequency ultrasound, with or without the use of energy-based modalities on the same day, in a real-world aesthetic practice.

Methods

This retrospective, single-center observational study included 246 patients who underwent facial thread lifting with APTOS Excellence Visage sutures under real-time ultrasound guidance. A portion of the patients received same-day energy-based treatments (linear focused ultrasound, monopolar radiofrequency, or CO₂ laser). Safety outcomes were assessed over 180 days. All patients were analyzed as a single cohort.

Results

The overall incidence of adverse events was below 5%, with no serious or irreversible complications. Reported events included self-limited postoperative pain (4.1%), ecchymosis (2.0%), hematomas (0.8%), and granulomas (1.6%). No cases of infection, thread extrusion, allergic reaction, or thread removal were observed. Ultrasound guidance confirmed accurate SMAS-plane placement in all procedures.

Conclusions

Thread lifting with PLLA/PCL sutures demonstrated a favorable safety profile when performed under AI-assisted ultrasound guidance. The use of real-time imaging enhanced anatomical precision. The combination of threads with same-day energy-based modalities was also safe, with no increase in complication rates.

## Introduction

Facial aging is characterized by progressive loss of skin elasticity, descent of soft tissues, and structural weakening of the ligamentous and fascial support system [1]. In recent decades, barbed suspension threads have emerged as a promising alternative to facial rejuvenation with minimal downtime. Since their first introduction by Sulamanidze et al., antiptosis threads have demonstrated safety and efficacy in thread lifting [2,3]. Absorbable threads composed of poly-L-lactic acid (PLLA) and polycaprolactone (PCL) act via a dual mechanism of action, combining immediate mechanical repositioning and long-term biostimulation via neocollagenesis [3-6].

The correct anatomical plane of thread placement, particularly within the superficial musculoaponeurotic system (SMAS), is essential for optimal and lasting outcomes [3,7,8]. Real-time high-frequency ultrasound has been proposed as a valuable adjunct, allowing visualization of facial fat compartments, ligaments, vessels, and the SMAS plane during cannula advancement and guide thread implantation, potentially minimizing risk [9,10].

Beyond intraoperative guidance, ultrasound also plays an important role in the diagnosis and management of complications. High-resolution ultrasound permits identification of thread position, detection of nodules, granulomas, or fistulas, and assessment of inflammatory activity, guiding appropriate interventions [11]. This dual role, preventive during implantation and diagnostic in complication management, has positioned ultrasound as an important tool in aesthetic practice [12].

Another area of growing clinical interest is the combination of suspension threads with energy-based technologies, such as focused ultrasound, radiofrequency, and ablative lasers. These modalities remodel dermal and subdermal tissues, complementing the mechanical support of threads. Early clinical evidence suggests that such associations are safe and may even reduce inflammatory complications when applied with proper timing and anatomical control [13]. Nevertheless, robust real-world data on the safety of these combinations remains scarce.

This study provides a real-world safety evaluation of thread lifting procedures with APTOS PLLA/PCL sutures (APTOS Excellence Visage®; APTOS LLC, Tbilisi, Georgia) placed under artificial intelligence (AI)-assisted high-frequency ultrasound, both as a standalone procedure and in combination with energy-based technologies.

## Materials and methods

Study design

This retrospective, single-center observational study evaluated the safety of 246 consecutive patients who underwent minimally invasive facial thread lifting using barbed absorbable PLLA/PCL sutures with or without association to energy-based technologies at Olsen Almeida Dermatology, São Paulo, Brazil, between November 2024 and May 2025. All thread implantations were performed under real-time ultrasound guidance with AI-assisted layer segmentation, by the same physician with expertise in minimally invasive aesthetic techniques using a standardized protocol. The primary endpoint was the safety of ultrasound-guided thread lifting with APTOS sutures. A secondary, exploratory analysis compared complication rates between patients who underwent thread lifting alone and those who received same-day energy-based treatments.

The use of energy-based technologies, including linear intensity focused ultrasound (LIFU), bulk heating technology, monopolar radiofrequency, and ablative CO2 laser, is routinely applied in our clinic, associated with thread placement, prior to its insertion, following standardized parameters. This retrospective study was conducted using anonymized chart reviews, with no patient contact or collection of identifiable information. As such, it was considered exempt from formal Institutional Review Board approval. All procedures complied with the ethical principles outlined in the Declaration of Helsinki.

Patient selection

Eligible participants were adults presenting with mild to moderate facial skin laxity treated with APTOS threads. Patient data were collected retrospectively from an institutional electronic health record database, and standardized scales of laxity were not systematically applied.

Materials and devices

All procedures employed the APTOS Excellence Visage thread, composed of a co-polymer of PLLA and PCL, 19 cm in length. Threads were implanted following standard technique with entry points and vector planning individualized to each patient’s anatomy. Energy-based modalities, when used, were applied on the same day, prior to thread implantation, in accordance with the clinic protocols and included high-intensity focused ultrasound (LinearZ®; Jeisys Medical, Seoul, South Korea), monopolar radiofrequency (Density®; Jeisys Medical), and a CO₂ laser device (Ultra Pulse®; Lumenis Be Ltd, Israel).

Ultrasound guidance was performed using a wireless high-frequency 15 MHz linear probe (Clarius L15 HD; Clarius Mobile Health Corp., British Columbia, Canada). Real-time anatomical visualization was supported by the T-Mode™ AI software (Clarius Mobile Health Corp.), enabling dynamic differentiation of subcutaneous fat, the SMAS, and deep fat compartments, with additional Doppler mode activation to avoid vascular structures during cannula navigation.

Procedure technique

Each treatment was planned to follow individualized lifting vectors, based on the anatomical distribution of retaining ligaments and areas of tissue descent, and following standard APTOS methods.

**Figure 1:**
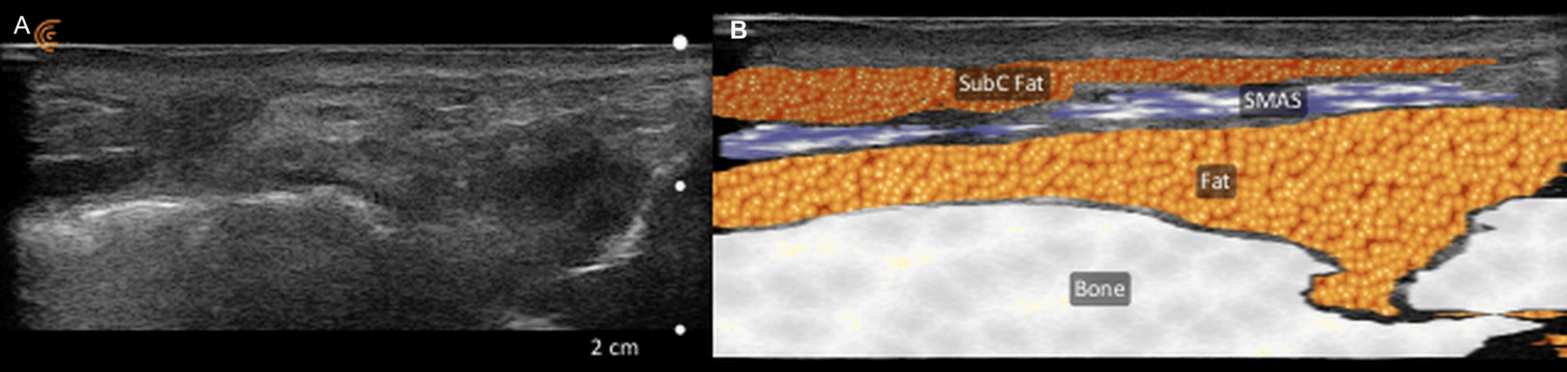
High-frequency ultrasound image with AI-guided tissue segmentation used during thread lifting procedure. (A) Real-time ultrasound identifying fascial and fat layers. (B) AI-generated segmentation: subcutaneous fat (“SubC Fat”), the superficial musculoaponeurotic system (SMAS), deep fat (Fat), and underlying bone. The thread cannula was advanced in real time within the SMAS plane under imaging guidance. AI: artificial intelligence

With the patient in the upright position, entry and vector points were marked, and the procedure was performed with the patient in the supine position. Antisepsis was performed, and sterile surgical drapes were positioned. Entry points were infiltrated with 1% lidocaine containing epinephrine, and the thread's path, previously marked, was also infiltrated with an anesthetic with a cannula. Implantation was carried out using a blunt-tip microcannula under ultrasound guidance with Doppler mode. This approach allowed anesthetic delivery while avoiding major vessels, particularly in the scalp (perpendicular to the pupillary line), zygomatic, and submandibular entries (1 cm below the mandibular angle). An illustrative example of this AI-guided segmentation is presented in Figure 1.

The threads were implanted in the upper third, midface, and/or submandibular region, and the number of threads was individualized according to regional requirements. Thread trajectories were planned based on anatomically defined lifting vectors. Cannula advancement was continuously monitored using a wireless ultrasound probe placed inside a sterile cover to maintain asepsis. Real-time ultrasound guidance was used during the procedure. This imaging modality allowed continuous monitoring of the cannula path and confirmation of thread placement in the SMAS layer, reducing the risk of malposition and potential vascular or neural injury [9-11]. Hemostasis was achieved through direct compression, followed by the application of occlusive dressings.

Safety outcomes were assessed retrospectively based on adverse events documented in electronic medical records over a 180-day follow-up period. Events were classified according to clinical severity and resolution. Although real-time ultrasound guidance was employed during all procedures to assist with anatomical navigation and plane verification, no ultrasonographic data were collected as predefined endpoints. Ultrasound was also selectively used to investigate suspected complications, such as granulomas or misplacement, when clinically indicated. No aesthetic or functional efficacy measures were systematically recorded.

Data analysis

Data were analyzed using descriptive statistics. Continuous variables were expressed as mean and standard deviation, and categorical variables as absolute and relative frequencies. Comparisons were performed using the chi-square test and Student’s t-test, as appropriate. A p-value < 0.05 was considered statistically significant. Analyses were performed with IBM SPSS Statistics for Windows, version 26.0 (IBM Corp., Armonk, NY, USA).

## Results

A total of 246 patients underwent facial thread lifting with APTOS Excellence Visage PLLA/PCL threads (19 cm length), guided by real-time high-frequency ultrasound with AI-based anatomical segmentation. Among these, 195 patients (79.3%) received energy-based skin preconditioning, including linear intensity focused ultrasound (LIFU), monopolar radiofrequency, or ablative CO₂ laser, immediately prior to thread implantation, while 51 patients (20.7%) underwent thread lifting alone. For safety analysis, the entire cohort was evaluated as a single population, reflecting a real-world safety evaluation in aesthetic clinical practice and maximizing statistical power. An exploratory subgroup analysis was additionally performed to compare complication rates between patients treated with threads alone and those receiving same-day energy-based modalities.

The cohort consisted of 211 women (85.8%) and 35 men (14.2%), with a mean age of 47.9 ± 8.6 years. No statistically significant differences in age or sex distribution were observed between patients with or without energy-based preconditioning (p > 0.05).

The overall incidence of adverse events was low, with no serious or irreversible complications recorded over the 180-day follow-up period. Ecchymosis was reported in 2.0% of patients (n = 5), hematomas in 0.8% (n = 2), and self-limited postoperative pain in 4.1% (n = 10). Four cases of granulomas were observed (1.6%, n = 4), all of which resolved with conservative treatment or minor outpatient excision. No events of infection, allergic reaction, thread extrusion, or removal were reported. Table 1 summarizes the adverse events recorded in the total cohort.

**Table 1:** Adverse events reported up to 180 days post-procedure in the total cohort (N = 246)

Adverse event	Number of cases	Incidence (%)
Ecchymosis	5	2.0
Hematomas	2	0.8
Granulomas	4	1.6
Local Infection	0	0.0
Allergic Reaction	0	0.0
Thread Removal Required	0	0.0
Self-limited Postoperative Pain	10	4.1

A statistically significant difference was found in the occurrence of granulomas, which were more common in the thread-only group (3/51; 5.9%) compared to patients who received adjunctive energy-based treatments (1/195; 0.5%; p = 0.029). Other adverse events did not differ significantly between subgroups. All granulomas were successfully treated, two with intralesional triamcinolone and hyaluronidase and two with minor surgical excision under local anesthesia. Other events, such as transient postoperative discomfort, were mild and self-limited, resolving without the need for medical intervention.

Ultrasound guidance confirmed accurate thread placement within the SMAS layer in all procedures, with no observed cases of malposition or vascular compromise.

## Discussion

This real-world safety evaluation of 246 facial thread lifting procedures using APTOS PLLA/PCL sutures under AI-assisted ultrasound guidance demonstrated a low complication rate (<5%) and no serious or irreversible adverse events. These results support the feasibility and safety of thread lifting in routine aesthetic clinical practice, particularly when performed under direct anatomical visualization.

Across the cohort, ecchymosis (2.0%), self-limited pain (4.1%), hematomas (0.8%), and granulomas (1.6%) were the only adverse events observed, all of which resolved without long-term sequelae. The absence of complications such as infection, vascular injury, extrusion, or thread removal is noteworthy and compares favorably with complication rates reported in prior meta-analyses. Niu et al. (2021), for example, reported pooled rates of ecchymosis (7.7%), skin dimpling (10%), and pain (11.8%), underscoring the importance of procedural standardization and advanced imaging in reducing risks [14].

Real-time ultrasound guidance with AI-assisted segmentation likely played an important role in mitigating complications. By enabling continuous visualization of the cannula trajectory, SMAS, vascular landmarks, and fascial layers, ultrasound facilitates precise placement of suspension sutures and reduces the likelihood of vascular, ductal, or neural injury. This observation is consistent with previous reports, such as Lee et al. (2020), who demonstrated that Doppler ultrasound-guided thread lifting avoided frontal branch injury of the superficial temporal artery [10], and Kim et al. (2023), who reported improved detection of parotid duct injury and more accurate management of complications with ultrasound navigation [9]. Other studies have further emphasized its value for verifying anatomical planes, identifying customized danger zones, and locating critical structures during thread lifting [7,15]. High-resolution ultrasound has also been shown to be effective in characterizing nodules, granulomas, fistulas, and thread malposition, enabling tailored treatment strategies [9,11]. Beyond these established benefits, the incorporation of artificial intelligence represents an important evolution. Haykal et al. (2025) highlighted that AI-enhanced ultrasound can reduce operator variability, standardize imaging protocols, and provide real-time decision support in aesthetic procedures [16]. Similarly, Tenajas et al. (2023) demonstrated that AI assistance can elevate the performance of less experienced clinicians to levels approaching expert operators [15]. These insights suggest that AI-assisted layer segmentation, as applied in the present study, not only contributed to accurate SMAS placement and lower complication rates but also holds potential to make thread lifting safer and more reproducible across different levels of practitioner expertise.

While the primary objective of this study was to assess safety independent of associated technologies, an exploratory analysis revealed a statistically lower incidence of granulomas in patients who received energy-based preconditioning (0.5%) compared to those treated with threads alone (5.9%; p = 0.029). Although this was not powered for subgroup analysis, these findings suggest a possible role of thermal remodeling in modulating local tissue inflammatory response and facilitating thread integration. However, this hypothesis remains speculative and warrants confirmation through prospective, controlled studies.

The combination of threads with energy-based modalities is an evolving area of interest. Casabona (2020) evaluated the association of suspension threads with microfocused ultrasound with visualization (MFU-V) and found no increase in adverse events or thread degradation, suggesting that when applied sequentially, the combination is clinically safe [17]. The present clinical study showed no increased inflammatory or vascular complications in patients who underwent thread lifting following energy-based treatments, reinforcing the safety of this combination. Miranda proposed a clinical protocol combining polydioxanone (PDO) threads with ablative and non-ablative technologies in aesthetic medicine, although evidence remains preliminary and lacks large-scale validation [13].

Taken together, these findings suggest that APTOS thread lifting is safe both as a standalone intervention and when used after energy-based preconditioning, provided that technique, timing, and anatomical control are respected. The systematic use of high-resolution ultrasound with AI segmentation may be reassuring for safety-consistent outcomes.

Limitations of the present study include its retrospective and unicentric design, absence of standardized efficacy metrics (e.g., Global Aesthetic Improvement Scale (GAIS), Wrinkle Severity Rating Scale (WSRS)), and short follow-up limited to 180 days. The retrospective nature limited the ability to systematically collect certain data, such as detailed ultrasound parameters, while the single-center design reduced external generalizability. The absence of standardized aesthetic endpoints restricted the scope of conclusions to safety rather than efficacy. Although procedures were performed by an experienced injector, the lack of inter-operator variability precludes assessment of reproducibility across different practitioners. Finally, while the 180-day follow-up provides relevant medium-term safety data, longer observation would be required to fully assess late-onset adverse events or the durability of outcomes.

Nonetheless, this study provides important real-world evidence on the safety of thread lifting with modern sutures and advanced imaging. It also establishes a foundation for future research and underscores the need for prospective, controlled trials with objective outcome measures to validate multimodal protocols involving energy-based devices.

## Conclusions

This real-world observational study demonstrated that facial thread lifting with APTOS PLLA/PCL sutures is a safe procedure when performed using a standardized technique in a clinical aesthetic setting. Across 246 cases, the overall complication rate was below 5%, with all events being mild and self-limiting. The integration of real-time ultrasound was decisive in confirming SMAS-plane placement and preventing vascular or neural injury, supporting its role as a procedural standard.

Furthermore, a large proportion of patients underwent same-day energy-based treatments with no increase in overall adverse events in this subgroup, including a statistically lower incidence of granulomas compared to the threads-only group. Taken together, these findings suggest the importance of advanced imaging in improving safety and consistency, while also supporting the safe application of multimodal protocols. This study provides a foundation for future research and underscores the need for prospective, controlled trials to validate long-term outcomes and innovative, combined approaches in minimally invasive facial rejuvenation.
